# Effective World Modeling: Multisensor Data Fusion Methodology for Automated Driving

**DOI:** 10.3390/s16101668

**Published:** 2016-10-11

**Authors:** Jos Elfring, Rein Appeldoorn, Sjoerd van den Dries, Maurice Kwakkernaat

**Affiliations:** 1 Integrated Vehicle Safety department, Netherlands Organization for Applied Scientific Research TNO, Helmond 5700 AT, The Netherlands; rein.appeldoorn@tno.nl (R.A.); maurice.kwakkernaat@tno.nl (M.K.); 2 Department of Mechanical Engineering, Eindhoven University of Technology, Eindhoven 5600 MB, The Netherlands; svddries@smart-robotics.nl

**Keywords:** multisensor, data fusion, world modeling, automated driving

## Abstract

The number of perception sensors on automated vehicles increases due to the increasing number of advanced driver assistance system functions and their increasing complexity. Furthermore, fail-safe systems require redundancy, thereby increasing the number of sensors even further. A one-size-fits-all multisensor data fusion architecture is not realistic due to the enormous diversity in vehicles, sensors and applications. As an alternative, this work presents a methodology that can be used to effectively come up with an implementation to build a consistent model of a vehicle’s surroundings. The methodology is accompanied by a software architecture. This combination minimizes the effort required to update the multisensor data fusion system whenever sensors or applications are added or replaced. A series of real-world experiments involving different sensors and algorithms demonstrates the methodology and the software architecture.

## 1. Introduction

Automated driving is gaining the interest of car manufacturers, media and the general public. Both the number of Advanced Driver Assistance Systems (ADAS) and the complexity of these ADAS are increasing [1]. Cruise control has been around for decades; more recent ADAS entering the market include adaptive cruise control with stop and go functionality, automated parking, traffic jam assist, automated emergency braking and Tesla’s AutoPilot.

The consequence of the increasing complexity of ADAS applications is an increasing number of sensors to monitor a larger part of the environment around the vehicle. Another reason for increasing the number of sensors is redundancy required to ensure safe operation. Furthermore, multisensor setups enable combining different sensor modalities, each with their own strengths. Increasing interest for cooperative driving [2] and V2x communication leads to even more diversity in input data for automated vehicles.

The configuration of the increasing number of sensors on vehicles evolves with the development of new applications and the introduction of new sensors on the market. This complicates implementing generic multisensor data fusion algorithms that take all sensor data and compute a world model that can be used for various applications. Tailored multisensor data fusion architectures that have to be completely redesigned and re-implemented each time a sensor or application changes are costly and undesirable. A one-size-fits-all multisensor data fusion architecture that is able to serve all possible applications is unrealistic given the large diversity in vehicles, sensors and requirements imposed by different applications. The world model computed by the multisensor data fusion algorithm can for example be as simple as a single point mass with a position and a velocity or as complex as a 360 degree world model including a precise host vehicle state estimate and many vehicles and road users each with their own kinematics and 3D geometrical description. Furthermore, safety critical applications, such as automatic emergency braking or cooperative adaptive cruise control, prefer low latencies over smooth signals, whereas the contrary may be true for lane-keeping assistance systems.

This paper is about constructing a world model by fusing information from all sensor data available. As an alternative for a one-size-fits-all multisensor data fusion architecture, this work introduces a multisensor data fusion methodology. It makes design decisions and dependencies between the various components explicit and suggests families of suitable algorithms. The methodology is derived after giving a broad overview of relevant literature in the context of automated driving. The overview helps those less familiar with the field understand both the challenges and possible solutions. As a second contribution, a modular multisensor data fusion software architecture that is compatible with the methodology’s philosophy is introduced. Designing multisensor data fusion software architectures is a non-trivial task that is heavily underexposed in the automated driving literature. Reusable software arguably is as important as reusable algorithms and hardware, especially in the research and development phase. The combination of methodology and software architecture offers a way to quickly update or reconfigure an existing multisensor data fusion architecture. This way, the required effort needed to redesign and re-implement the fusion component in case of unforeseen changes in applications, requirements or sensors is minimized. As a third contribution, the methodology is demonstrated by applying it to a diverse set of real-world applications involving different sensors, algorithms and applications. Throughout this work, the focus lies on world models at the ‘object level’. Integrating such world models with, e.g., navigation maps, is left for future work.

This paper is organized as follows. Section 2 summarizes related work with an emphasis on automated driving-related problems; Section 3 introduces the multisensor data fusion methodology, and Section 4 introduces the accompanying software architecture; Section 5 explains implementation-related details, and Section 6 demonstrates the proposed methodology and software architecture using a series of real-world examples; finally, Section 7 summarizes the conclusions.

## 2. Related Work

World modeling in automated driving is usually solved by adopting state estimation techniques that fuse information from one or more sensors over time. State estimation is actively being researched for more than half a century, and in this section, an overview of the most important works is presented. The overview: (i) starts with a broad application independent overview and then focuses on the domain of automated driving; (ii) only considers methods that have their foundations in probability theory, since these are considered the standard approach [3] in the domain; and (iii) leaves out mathematical details not needed for getting the bigger picture.

For those unfamiliar with Bayesian filtering, we refer to related works, like, e.g., [4], for a more detailed problem definition and a more elaborate and mathematical introduction into the problem of Bayesian filtering. An elaborate and application independent survey on the more generic problem of multisensor data fusion can be found in [5].

### 2.1. Single Sensor State Estimation

The Bayesian approach [3] to state estimation recursively applies a prediction and update step to refine an estimate of some state as new measurement data arrive. The prediction step uses a system model to predict how the state evolves over time, whereas the update step refines the predicted state estimate by incorporating knowledge obtained via measurements. Finding the optimal solution to the recursive Bayesian filtering problem in general is intractable due to intractable integrals in the prediction and update steps. By assuming, among others, linear models and known Gaussian noise distributions, the integrals can be solved analytically, and the exact minimum mean square error solution is described by the well-known Kalman Filter (KF) [6]. Despite its firm assumptions, the KF has proven to be extremely useful and has been successfully applied many times, e.g., [7]. A linear mapping from the state space (what we would like to estimate: for example, the kinematics of traffic participants) to the measurement space (what we measure: for example, a radar’s reflected radio waves, a camera image or a LiDAR’s point cloud) often does not exist. Therefore, the traditional approach to single sensor state estimation, usually first summarizes sensor data by features that have a known linear relation with the state space and then applies the Kalman filter. Possible implications of the feature extraction step will be discussed later.

In case the models involved are nonlinear and noise is still known and Gaussian, suboptimal solutions have been derived from the optimal Kalman filter. The Extended KF (EKF) [8] uses a Taylor expansion to approximate the nonlinear models, and the Unscented KF (UKF) [9] uses the unscented transform for the nonlinear transformation of the Gaussian distributions. Nonlinear models may turn Gaussian distributions into non-Gaussian distributions. Furthermore, due to the approximations involved, the estimates might be biased or diverge; however, the EKF and UKF have proven to be very useful, e.g., [10,11]. When applying a (E/U)KF, a data association step that associates measurement data with new or previously-observed objects or false alarms is essential, since incorrect associations may lead to large estimation error and diverging filters. Whenever we refer to tracks, we refer to the output of any estimator introduced in this section.

**Figure 1:**
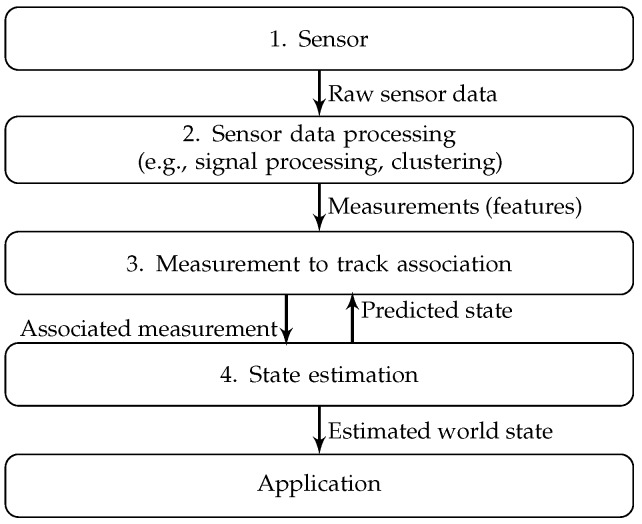
Traditional single sensor state estimation pipeline.

Figure 1 summarizes what we refer to as the ‘traditional approach’, where one (1) collects data from a single sensor and (2) summarizes these data into features (processed sensor data, e.g., a vehicle detected in the camera image); then, these features are (3) associated with tracks estimated using state estimators, and (4) single sensor state estimators are updated using the associated measurements. Much of the current work in the domain of automated driving is inherited from this approach and focuses on one of the steps in the pipeline, e.g., sensor data processing [12], state estimation [13] or data association [14].

The pioneering work of [15] led to an alternative Bayesian state estimator: the Particle Filter (PF). Many flavors exists [16]; however, they all represent the estimated state by a set of weighted particles. Each particle represents a possible value for the state and the posterior distribution (the state estimate after incorporating all measurement data available) is represented by the set of particles. Particle filters do not constrain the models involved and are able to represent probability distributions of arbitrary shapes in case a sufficient number of particles is used. As the number of particles increases, the estimates of the intractable integrals converge to the true values; hence, the PF estimate converges to the optimal solution in case the associated models are correct. Recent works exploiting different kinds of PFs for automated driving include [17,18,19]. One of the main drawbacks of PFs is the exponential scaling of the number of required particles with the dimension of the state space. A popular way to reduce the computational complexity is adapting the number of particles during estimation based on the focus of the posterior [20]. Another widely-applied technique is reducing the size of the state space associated with the particles by analytically marginalizing out substructures, which are tractable conditional on other parts of the state space [21] (Rao-Blackwellization). A computationally-attractive alternative to particle filters in high dimensions are Markov chain Monte Carlo algorithms [5]. Due to its ability to handle arbitrary nonlinear models, the particle filter offers the option of dropping the sensor data processing step in Figure 1 and may simplify the data association step, e.g., as in demonstrated in [19].

Finite-Set Statistics (FISST) offers a way to perform multiple object tracking without an explicit measurement-to-track association step [22]. One probability distribution on a finite set variable conditioned on the time-history measurement-set is constructed, and a recursive Bayes filter is applied without the need for data association. Approximations like the Probability Hypothesis Density (PHD) filter [23], the cardinalized PHD filter [24], the Gaussian Mixture PHD (GM-PHD) filter [25] or the Sequential Monte Carlo implementation of the Cardinality-Balanced Multi-Target Multi-Bernoulli (SMC-CBMTMB) filter [26] are common ways to render the filter computationally tractable. The specific implementation of these filters determines whether (e.g., SMC-CBMTMB) or not (e.g., GM-PHD) nonlinear models can be facilitated. Extensions like [27] allow for consistent track identities over time.

### 2.2. Multisensor Data Fusion

As explained in the Introduction, automated vehicles are multisensor systems. We start by considering Figure 1. In case the goal is fusing information from different sensors, this can be done combining the information at either one of the following levels:

1. Low-level sensor data: unprocessed measurement data or features extracted from sensor data are fed to a fusion algorithm
2. Tracks estimated by single sensor state estimators are fused.

#### 2.2.1. Multisensor Low-Level Sensor Data Fusion

One approach is fusing sensor data at a low level. A precise definition of ‘low-level sensor data’ is outside the scope of this work, and therefore, the distinction between low- and high-level data will be somewhat subjective. In this work, low-level data refer to sensor data that did not undergo any temporal filtering, such as averaging or tracking.

Fusing low-level information can be performed using a Bayesian state estimator, e.g., some sort of Kalman or particle filter, that takes measurement data collected by different sensors. Each sensor has its own characteristics; hence, multiple measurement models relating the state space to the measurement space will be needed. Another common way of performing low level sensor data fusion is to first fuse all sensor data (sometimes referred to as measurement fusion) and then use a single Bayesian estimator with a single measurement model. One advantage of low level sensor data fusion is the ability to (indirectly) exploit information from one sensor while interpreting sensor data from another sensor. This, in general, improves both the algorithm’s robustness and performance [28]. In addition, sensor data processing is equivalent to sensor data reduction. Low-level sensor data fusion therefore minimizes the information loss [29]. Furthermore, it reduces the risk of adopting inconsistent modeling assumptions while processing data from different sensors [30].

A drawback is the need to transfer an increased amount of data from the sensor to the platform that runs the estimation algorithms. This imposes heavier constraints in terms of communication loads. Furthermore, feeding lower level data to the state estimation algorithm requires more advanced measurement models and a thorough understanding of the low-level sensor data. Low-level sensor data fusion is used in, among others, [31,32,33].

#### 2.2.2. Multisensor High-Level Sensor Data Fusion

In case the sensor data are being processed by a state estimator that includes temporal filtering, we refer to the data as high-level sensor data. Typically, the data now are at the ‘tracked object level’. Tracked object level data delivered by different filters often are correlated, e.g., due to shared modeling assumptions [34], the double counting problem [35], common noise acting on the object being tracked [36] or measurements arriving out-of-sequence [37]. Many Bayesian filters assume uncorrelated input data and may deliver overconfident or diverging estimates in case such correlations are ignored [4,5]. A different family of algorithms was developed in the last decades to handle exactly these kinds of problems: track-to-track fusion algorithms.

Track-to-track fusion algorithms can roughly be divided into three groups. The first group solves the track-to-track fusion problem in its most general form by assuming no prior knowledge on the correlations between the tracks. The Covariance Intersection (CI) algorithm is the most popular method in this first group of algorithms [36]. It is proven to deliver consistent estimates, but involves an optimization step that might be computationally demanding and leads to conservative fused estimates [38]. Among the faster, but for some situations, worse performing, alternatives are, e.g., the fast CI algorithm [39] and the improved fast CI [40] algorithm. Among the less conservative alternatives are the largest ellipsoid [41] and the internal ellipsoid approximation [42] algorithms. Most of these methods cannot prove that the fused estimate has an estimation error covariance matrix that is equivalent to or smaller than the original estimate’s accuracy. An exception is the Ellipsoidal Intersection [43] (EI) algorithm. The ellipsoidal intersection is likely to be consistent for real-world applications; however, consistency cannot be proven.

A second group calculates the correlations. The Information Matrix Fusion (IMF) algorithm [44] is the most popular algorithm, and it is successfully applied for automated driving in, e.g., [45]. It handles the unknown correlations by memorizing individual measurements and then only fuses new information by de-correlating each incoming track. An alternative is calculating the cross covariance as proposed by the Cross-Covariance Method (CCM) [46]; however, this requires communicating more than just object-level measurements, e.g., Kalman filter parameters. Whether or not these options are feasible depends on the sensor topology and communication abilities. For dynamic network configurations, e.g., in cooperative driving with many ad hoc communication networks, IMF is not possible in general. The cross-covariance method is not feasible for black box sensors that do not expose internal filter settings or systems with strict communication constraints; however, methods to approximate the cross-covariance do exist [47].

The third and last group of track-to-track fusion algorithms assumes the correlations to be known. In case correlations are fully known, the Best Linear Unbiased Estimate (BLUE) can be used [48]. The CCM of [46] or either one of the methods proposed in [49] are some of the available methods that can be used in case correlations are partially known. Kalman filter-based solutions typically fall into either the second or third group [37]. Exploiting knowledge on correlations (as is done in the third group) or approximating correlations (as is done in the second group) improves the estimation accuracy compared to solutions in the first group, but is not always realistic.

One advantage of high-level sensor data fusion, compared to low-level sensor data fusion, is the limited amount of data that must be transferred due to the hierarchical structure of the estimation framework. In addition, many popular automotive sensors apply onboard processing and only deliver data at the tracked object level. Interpreting the measurement data is simpler and no longer requires in-depth knowledge on the working of the sensor or the signal processing algorithms involved. Furthermore, using object-level data leads to more homogeneous processor loads [30] and enables standardizing the interface towards the fusion algorithm. This simplifies replacing sensors or the sensor topology without changing the fusion algorithm.

Important drawbacks are the exact opposite of the advantages of the low-level sensor data fusion: potentially useful information is discarded before the fusion takes place, and one can in general not exploit information from one sensor while interpreting data from another sensor. Furthermore, application-specific preferences, e.g., on the accuracy or latency of data, can barely be affected by tuning the fusion algorithm, since many decisions affecting these characteristics were already being made while designing filters attached to sensors. Furthermore, many of the track-to-track fusion algorithms are covariance matrix based and therefore are not directly applicable to fusing non-Gaussian distributions, e.g., delivered by particle filters or for fusing data from sensors that do not deliver an error covariance matrix.

High-level sensor data fusion arguably is the most popular way of multisensor data fusion for automated driving and is adopted in [34,50,51,52]. One can use track-to-track fusion algorithms for low-level sensor data fusion as long as the data are of the appropriate form. Low-level sensor data fusion is not suitable for fusing high-level object data for the reasons given before.

### 2.3. Data Association

Incorrect data association leads to increased estimation errors or even diverging estimates and is crucial for the overall multisensor data fusion performance. Besides assigning measurements to tracks associated with real-world objects, often dummy tracks are used to handle false positives. This section gives a very brief overview of the field whereas, e.g., [53], gives a more complete and detailed overview.

One of the most popular methods is the Multiple Hypothesis Tracking (MHT) filter [54,55]. It enumerates all possible solutions to the association problem, referred to as hypotheses, and computes probabilities for each of these. As a result, MHT is both exponential in time and memory. For that reason, all but a limited number of most probable hypotheses are pruned at each time step. Maintaining multiple hypotheses allows for revising (correcting) previous association decisions based on new evidence, which makes MHT “the preferred method for solving the data association problem” according to [56]. Two popular data association alternatives include the Global Nearest Neighbor (GNN) filter [57] and the Joint Probabilistic Data Association Filter (JPDAF) [58]. In case one only keeps the most probable hypothesis, the GNN results can be obtained from the MHT filter, whereas the JPDAF results are obtained by probabilistically weighting a subset of hypotheses and pruning all others. Both the GNN filter and the JPDAF require more recent advances, e.g., [59], to handle a varying number of objects.

### 2.4. Multisensor Data Fusion for Automated Driving

Multisensor data fusion in the domain of automated driving differs from the classical tracking problem in a number of ways. This section focuses on some of these aspects.

#### 2.4.1. Object Existence

The probability of existence is an important characteristic for each of the objects in automated driving applications. Unnecessary automatic emergency braking or evasive maneuvers are unacceptable and might lead to accidents. Overlooking objects might also have severe consequences.

The majority of the above-mentioned works exclude track management; however, some methods handle this problem in a natural way. The Bayesian MHT filter framework for example includes probabilities for target birth and track termination [55], and joint integrated probabilistic data association adds the target existence concept to the JPDAF. Alternatives include [60], where existence probabilities are used for track management and more heuristics-based approaches where tracks are initiated or terminated based on, e.g., (the absence of) consecutive updates [32].

#### 2.4.2. Object Representation

Many of the related works are developed for ‘point objects’ that are adequately represented by a unique identifier and a state vector representing position, velocity and acceleration. For automated overtaking, object dimensions are crucial, and for automated crossing of an intersection, the object type must be known, i.e., pedestrians and trucks move differently. For cooperative adaptive cruise control, other road user’s intended accelerations are shared, and for traffic lights, the color is important, whereas for bicycles, this typically is not. This work treats positions, velocities, accelerations and shapes of objects in a similar manner as the probability of existence, object class or color of a traffic light. These are all object-specific properties that must be estimated using appropriate state estimation algorithms and represented by appropriate, possibly different, probability distributions.

#### 2.4.3. Multisensor Data Fusion Architectures in Automated Driving

Generic multisensor data fusion architectures for automated driving include [30,33,50]. Each of these work addresses the issues in the sections above in a different way, and there is no consensus on which of the various options to select.

In [50], high-level (track-to-track) fusion methods are adopted mainly due to the modularity and the simplicity of the design. Furthermore, the authors state that the diversity of sensor types and locations complicates low level fusion. In [33], low (feature)-level fusion is favored instead since, according to the authors, track-to-track fusion “does not solve the sensor-independence in the track management functionality” and “does not provide any sensor independent measurement of the track quality”. The authors in [30] claim that it is beneficial to perform association at both levels. Both [33] and [50] adopt fixed interfaces to the multisensor data fusion algorithm, and both use Gaussian distributions for this. The measurements in [33] are accompanied by a spatial uncertainty represented by a covariance matrix and a probability of existence. The tracks in [50] contain position, velocity, acceleration, object length and width and a probability of existence. In [30], no interfaces are specified.

As a result, [33,50] complicate dealing with non-Gaussian states, e.g., the color of a traffic light, the intention of a communicating vehicle or any quantity that is associated with a multimodal probability distribution. In addition, these architectures enforce making at least some of the assumptions at design time despite the fact that applications and sensors may change over time. Assuming normal driving might be very reasonable for ACC, whereas adopting the same assumption for a collision avoidance application might be too conservative [30]. Furthermore, the trade-off between smooth estimates and low latencies will be very different for, e.g., a lane-keeping assistance function or cooperative ACC. Handling such contradicting requirements is beyond what is shown in [33] and [50]. The work of [30] only explains the conceptual level and does not offer ready-to-use solutions. None of the works incorporates software architectures despite the fact that a flexible architecture is only possible with flexible software and designing such architectures is non-trivial.

### 2.5. Related Work Conclusions

One fixed interface between all sensors and fusion algorithms for all applications is undesirable due to the varying requirements on the estimation accuracy, the type of information that must be estimated, the computation and communication resources and the communication topology. A one-size-fits-all multisensor data fusion architecture that offers optimal performance and only adopts assumptions that are reasonable for all applications is unlikely to exist.

As an alternative, this work introduces and validates a multisensor data fusion methodology that leads to the appropriate set of multisensor data fusion algorithms. The methodology, together with the overview in this and the following section, suggests and advises against algorithms and interfaces with the domain of automated driving in mind. Contrary to all works explained so far, this work in addition proposes a software architecture that can effectively be reconfigured or updated whenever applications or sensors change. The software architecture is optimized towards (re)configurability and, therefore, is particularly useful while developing, prototyping and testing automated driving applications.

## 3. Multisensor Data Fusion Methodology

**Figure 2:**
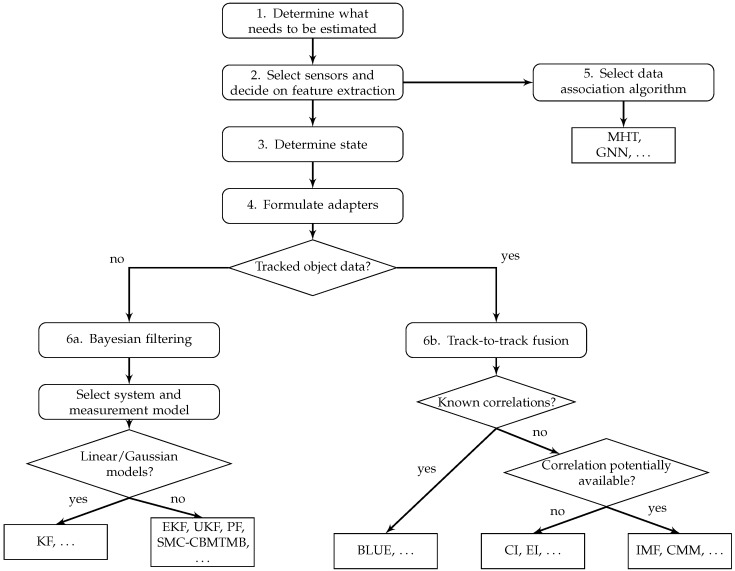
Block diagram showing the various steps in the proposed methodology. PF, Particle Filter; SMC-CBMTMB, Sequential Monte Carlo implementation of the Cardinality-Balanced Multi-Target Multi-Bernoulli; CI, Covariance Intersection; EI, Ellipsoidal Intersection; BLUE, Best Linear Unbiased Estimate; MHT, Multiple Hypothesis Tracking; GNN, Global Nearest Neighbor.

**Figure 3:**
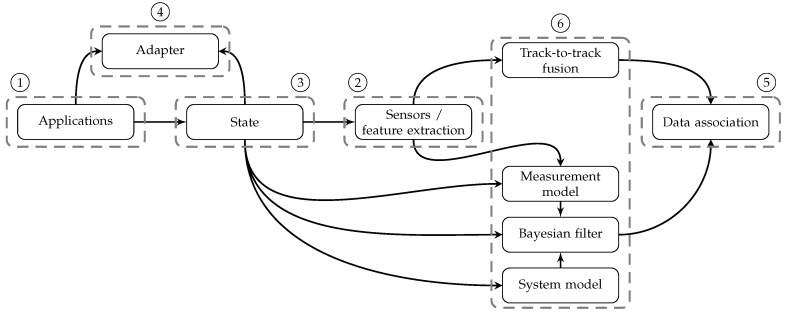
Constraints between decisions made in different steps of the methodology.

This section explains the proposed methodology for the design of a multisensor data fusion mechanism that is derived from the overview in Section 2. Figure 2 shows a graphical representation of the recommended work flow. Based on the specifics of the multisensor fusion problem at hand, the methodology suggests certain families of algorithms. For a more complete and detailed overview of suggested algorithms, the reader is referred to Section 2. Figure 3 indicates how decisions in one block impose constraints on the decision in another block. After introducing the methodology, Section 4 explains the proposed software architecture that is able to span the full range of possible solutions. Numbers in Figure 2 and Figure 3 refer to modules in the software architecture and represent different steps explained below.

### 3.1. Step 1: Application-Dependent Output Selection

The first step is to determine the data that are required for the application at hand. By selecting the desired output, the world model output (state) is constrained. Besides selecting what information is needed, it is also important to determine how this information is needed, e.g., a probability distribution representing a pedestrian’s kinematics and an associated probability of existence or just a position vector.

In case multiple applications must be served in parallel, the desired output to each of the applications must be considered. The algorithms can in this case be dependent (one fusion algorithm, hence minimal computational overhead) or independent (multiple fusion algorithms, hence no single point of failure). Section 5 on implementation will explain the options in more detail.

### 3.2. Step 2: Sensor Set and Feature Extraction

The second step is the selection of the set of sensors that will be used to generate the desired output. Besides the expected performance, cost and robustness, the availability of sensors plays an important role, e.g., complicating sensor data processing might be favored over adding a sensor. By selecting a sensor set, the input data for the world modeling algorithms are fixed, as well. The data can either be low- or high-level measurement data, as specified in Section 2.2. Communicated data, in this work, are interpreted exactly the same as low- or high-level sensor data from on-board sensors.

Many automotive sensors only deliver high-level (tracked) object data; hence, feature extraction is not needed. In case only low-level data are available, feature extraction must be considered depending on, e.g., the dimensionality of the data. Some automotive sensors offer the option to either use low- or high-level data. In case the engineer is familiar with the sensor data and knows how to interpret the data, using the low-level data potentially offers the best overall performance [28] at the expense of increased computation and communication requirements. In case the data are difficult to interpret, standard interfaces to the fusion algorithm are preferred, the communication bandwidth is limited or adequate feature extraction algorithms are available off-the-shelf, one should adopt feature extraction algorithms. An interesting example that compares the multisensor data fusion performance for both options in the context of simultaneous vehicle tracking and shape estimation using a LiDAR can be found in [19].

Feature extraction algorithms map low-level data into something that can be processed by estimation algorithm, e.g., a Gaussian distribution representing a measured vehicle position instead of a camera image or a bounding box representing the shape of a truck instead of a point cloud. Feature extraction requires computation and therefore adds delay to the chain from sensors to application. Feature extraction settings balance false positives (detecting something that is not there) and false negatives (not detecting something that is there). It is highly recommended to keep in mind the application-specific requirements on delays and false positives and negatives, while selecting and tuning these algorithms.

Among the most common feature extraction algorithms in automated driving are road, lane and vehicle detection. A complete overview of feature extraction algorithms is beyond the scope of this work; however, the surveys [61,62] can be starting points for interested readers. Recent advances on machine learning and deep learning [63,64], e.g., for object classification or detection, are categorized as feature extraction algorithms throughout this work.

The software architecture introduced later does not pose any restriction on the number of sensors, the type of sensors or the interface between the sensors and the multisensor data fusion algorithm and, therefore, allows for considering both technical and non-technical constraints in the sensor selection step.

### 3.3. Step 3: State Representation

The next step is to select the probability distributions that are most suitable for representing the state variables required for the desired world model output. The state vector may be a direct copy of the desired output; however, related variables can be added to improve the accuracy or simplify the estimation of the state. Knowledge on a vehicle’s dimensions for example may lead to a more accurate estimate of positions and velocities.

The expected shape of the probability distributions and possibly memory and computation resources determine which distributions can be used to represent the estimated values of these variables. Section 2 explained both the popularity and limitations of Gaussian distributions, e.g., the lateral position on the road typically follows a multimodal distribution in case one measures offset to ego-lane markings on a multi-lane road. Scalar probabilities are common for probabilities of existence and discrete probability mass functions represent discrete states such as color of a traffic light or object class. There is no single best state representation for all possible quantities, and therefore, the methodology and the software architecture later do not dictate one predefined state representation. Note that this does not imply that state representations cannot be reused. Furthermore notice that probabilistic state representation are always preferred, even in situations where the applications non-probabilistic information.

For the majority of applications multiple quantities will have to be estimated simultaneously, e.g., the positions and shape of lane markings, the kinematics of other road users, probabilities of existence and object types. It is important to decide whether or not these quantities are (assumed to be) dependent or independent. Dependent quantities, such as the position and velocity of the same vehicle, must be estimated by a single estimator. Whenever independence is a reasonable assumption, quantities must be separated since this lowers the computational costs without sacrifices on the estimation accuracy and enables parallel processing.

### 3.4. Step 4: Adapters

An application requires world model data in a specific format. Adapters take the full world state as formulated in Step 3 and convert them to the application-specific format defined in Step 1. Typically, adapters compute expected values and covariance matrices of the probability distributions representing states or round probabilities to binary numbers.

### 3.5. Step 5: Data Association

A data association algorithm decides which measurements and/or tracks originate from which object (if any). Incorrect data association might lead to diverging estimates and ghost or missed objects and, therefore, can have dramatic consequences for the quality of the estimated world model and performance of the application. The complexity of the data association algorithm may vary with the application and the measurements involved. From a data association perspective, tracking vehicles with relatively large inter-vehicle distances on a highway is simpler than tracking pedestrians in a crowded urban environment, and associating (high level) tracked object level data is simpler than (low level) dominant radar reflections. Furthermore, the characteristics of the feature extraction must be considered. Whenever false negatives must be avoided at all cost, the number of false positives will be higher, and the probabilistic data association models must be adapted accordingly. Section 2 already indicated the dependency between the method used for estimation and the need for data association.

### 3.6. Step 6a: State Estimation: Bayesian Filters

Based on the sensor output, either Bayesian filters or track-to-track fusion algorithms are recommended for state estimation. This section assumes Bayesian filters will be used, whereas Section 3.7 handles high-level state estimation using track-to-track fusion algorithms. Once again, the world modeling methodology does not force decisions since selecting estimation algorithms without knowledge on sensors or applications, as would be done by a one-size-fits-all world model, is undesirable for the reasons explained before.

#### 3.6.1. System Model

Bayesian filters contain a system model that represents prior knowledge on the state dynamics. Whenever the state is selected, a system model therefore describes how this state evolves over time given optional control inputs. System models are represented by conditional probability density functions [4], and some popular system models used in automated driving are explained in [65]. The system model and the associated uncertainty heavily affect the estimation results. In general a low system model uncertainty leads to estimates that emphasize the system model dynamics and typically are smooth. Large system model uncertainties make estimates more sensitive to measurements and typically less smooth.

Whenever system models are nonlinear, Gaussian state representation may turn into non-Gaussian distributions, and the state representation might need to be reconsidered. Section 2 discusses ways of dealing with nonlinear models in more detail, e.g., approximations like Taylor expansions.

#### 3.6.2. Measurement Model

The measurement model maps the predicted state to an expected measurement. In other words, the measurement model simulates a measurement (e.g., the position of a vehicle, the shape of a road marking, a full LiDAR scan) based on the state that is predicted using the system model. The mismatch between expected and actual measurement will later be used to update the estimate. The simulated measurement must be of the same type as the measurement delivered by the sensor or the feature extractor. By fixing the sensor set and the state variable, the measurement model’s input and output type are fixed. These constraints once more illustrate how a feature extraction step may simplify the formulation of a measurement model, e.g., the expected measurement may simplify from a full LiDAR scan to the position of a vehicle with respect the the coordinate system of the LiDAR. This may come at the expense of the estimation accuracy [19].

Measurement models in Bayesian filters are conditional distributions, and the associated uncertainty affects the characteristics of the estimation. Low measurement uncertainties represent more accurate measurements and lead to estimates that follow measurements closer. An increasing measurement uncertainty, like a decreasing system model uncertainty, puts more emphasis on the predictions, whereas the update step gains importance if the opposite holds.

The number of measurement models depends on the measurement, the number of independent states and the way of fusing sensor data.

#### 3.6.3. Bayesian Filter

The Bayesian filter performs the actual estimation and, together with the models defined in earlier steps, determines the performance of the estimation. The combination of state, system model and measurement model constrains the Bayesian filter as indicated in Figure 3 and explained in Section 2. In case multiple sensors are used to estimate the same state, a single Bayesian filter can be associated with multiple sensor-specific measurement models or the data can be fused before feeding it to the estimator.

### 3.7. Step 6b: State Estimation: Track-To-Track Fusion

In case the sensors involved deliver object track-level data, track-to-track fusion algorithms should be used for the estimation for reasons explained in Section 2. Track-to-track fusion algorithms do impose constraints on the shape of the distributions that will be fused. Which one of the algorithms is most suitable depends on the knowledge of correlations between the input data delivered by different sensors; see Figure 2 and Section 2. In case such prior knowledge is not available, the ability of estimating correlations further restricts the potential set of solutions as extensively explained in Section 2.

## 4. Architecture

The ideas underlying the methodology explained in Section 3 are used to design a compatible software architecture. First, Section 4.1 introduces the requirements posed on the software architecture and derived from the methodology. Then, Section 4.2 explains the architecture.

### 4.1. Requirements

Based on Section 2 and the proposed methodology explained in Section 3, a number of requirements that have to be met by any reusable and flexible world modeling software architecture are formulated. More general requirements on software architectures, e.g., separation of concerns [66], are outside the scope of this paper.

#### 4.1.1. Dependence versus Independence

Many applications require multiple quantities to be estimated, e.g., positions of road markings, kinematics of nearby objects, object classification, intended trajectories. As explained earlier, quantities may either be dependent on one another or not. Separating independent quantities in different estimators (1) increases the computational efficiency and (2) enables parallel processing. Combining dependent quantities in a single state estimator simplifies modeling the dependencies between these quantities. The software architecture should be able to facilitate both the estimation of dependent and independent quantities. Ideally, parallel estimation of independent quantities is possible for computational efficiency reasons.

#### 4.1.2. Interfaces

Sensor data fusion can be performed on various levels as explained in Section 2.2. Contrary to many of the sensor fusion works introduced in Section 2, the software architecture should neither prescribe the level at which sensor data fusion will be performed nor the data structure used to represent sensor data.

#### 4.1.3. Algorithms and Representation

The methodology leaves room for different ways of performing the state estimation and sensor data fusion. The same must hold for the software architecture. The dependencies between the various steps in the methodology, indicated by the arrows in Figure 2 must be respected and enforced by the software architecture. Furthermore representing the diversity of objects and their states as encountered in automated driving applications must be possible with minimal effort.

### 4.2. Software Architecture

**Figure 4:**
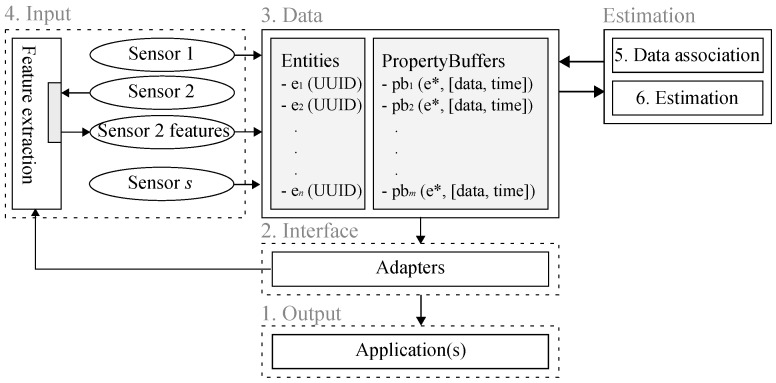
Proposed multisensor world modeling software architecture for a two sensor example. The numbers refer to the steps in the methodology that affect the various modules.

Figure 4 shows the proposed software architecture at a conceptual level. The architecture contains five main modules indicated with dashed lines and explained in this section. The numbers indicate which steps in the methodology affect the module. Section 5 explains the implementation level of the architecture.

#### 4.2.1. Data

The data module contains the actual world model. The world model represents data on the object level, and the decision on how to represent this data is one of the fundamental decisions underlying the software architecture.

An object-oriented decomposition of objects into a finite set of objects (classes) would be the most obvious choice, e.g., a vehicle object for dynamic objects from which trucks, buses and passenger cars derive their core properties and a traffic light object for static objects with a colored light state. The vehicle object would then have a kinematic state and a shape together with the appropriate update functions and a method for visualization. The traffic light would have a color and a position and, possibly some members related to the timing of the traffic light or methods to broadcast the current state to road users.

The main drawback of this approach is the complexity of maintainability due to a lack of flexibility. It must be possible to represent the kinematic state of vehicles with an arbitrary probability distribution as was explained in previous sections. The distribution can be discrete, e.g., a lane index as lateral position, or continuous, e.g., Cartesian coordinates with respect to an arbitrary coordinate frame. Furthermore, both the dimension and the semantics of the distribution may be different for different applications, e.g., Cartesian coordinates versus polar coordinates, 2D versus 3D positions or a state vector with or without acceleration term. A similar diversity exists for other properties, e.g., the shape of an object. To further complicate things, the underlying estimation assumptions on (in)dependencies may require the object’s kinematic and shape to be concatenated into one higher dimensional distribution or kept separately as two independent probability distributions. Finally, the set of possible properties is very large, and the subset of required properties is highly dependent on the application(s) at hand.

The decomposition of objects into a predefined object hierarchy would most likely lead to constantly shifting methods up and down the class hierarchy. The number of objects will increase while the ease of interpretation decreases, e.g., one object for a vehicle with dependent and one for a vehicle with independent kinematic state and shape. The regular introduction of new properties leads to overhead for older applications, and the length of source files grows while the maintainability gets worse.

As an alternative, the design in this work is inspired by the ‘composition over inheritance’ principle underlying the entity-component system paradigm [67]. This paradigm is gaining popularity in the game development domain and is, to the best of our knowledge, novel in the automated driving literature. Within the data module, objects are represented by entities. Entities can be anything present in the vehicle’s surroundings, e.g., other vehicles on the road, traffic lights, road markings or traffic signs, but it can also be the sensors on the host vehicle. Each entity contains a unique identifier. The identifiers are unique and time consistent handles to the various objects.

Besides entities, the data block contains property buffers that represent properties. Property buffers contain a pointer to a single entity and a cyclic buffer with time stamped data representing the property’s values over time. As a result, entities are unique; each property buffer refers to exactly one entity, and each entity can be associated with an arbitrary number of properties. Typical properties are, e.g., laser scan data associated with the laser scan entity or a position and velocity associated with a vehicle being tracked. Properties are usually, but not necessarily represented by probability density functions and adding new or changing existing properties can be done without affecting any of the other independent properties.

Separating independent properties into independent property buffers allows for parallel processing, even if properties belong to the same entity. Throughout this work, measurements are stored in cyclic property buffers pointing to specific sensor entities. This allows for reusing the same measurement in different algorithms running in parallel and possibly at different frequencies. Furthermore, it allows redoing estimations whenever this is desired, e.g., in case measurements arrive out-of-order.

#### 4.2.2. Input

The input module is responsible for adding measurement data to the world model. This information can be of any form, e.g., features, raw sensor data, tracked object data. In the example that is detailed in Figure 4, sensor data from Sensors 1 and *s* are directly provided to the world model, where they are added to a measurement property buffer pointing to the corresponding sensor entities. In addition, features extracted from Sensor 2 are added to the world model. Besides feature extraction, the input module also serves as a bridge between the sensor and the world model.

#### 4.2.3. Estimation

In the software architecture, the data are separated from the computation. The data blocks only represent data, whereas the input blocks add data from sensors. Constructing and maintaining a world model require executing estimation and track management algorithms. The estimation module may take any subset of world model data for performing the estimation and then feeds the results back to one or more property buffers. It roughly contains two modules: data association and estimation. Estimation can be performed by any track-to-track fusion or (Bayesian) state estimation algorithm explained in previous sections and includes, e.g., track management, probabilities of existence, classification and kinematic state estimation. The data association module takes sensor entities and associates the sensor’s data with compatible entities. If needed, entities can be removed or added. Data association is optional and can be on either the track or measurement level as long as the associated constraints are compatible.

The only requirements the architecture enforces here are the dependencies dictated by the methodology. Typically, modules in the estimation block subscribe to a specific property buffer and, whenever data are added, perform some computations and set the outcome into a different property buffer. A Kalman filter could for example subscribe to a ‘vehicle detection property buffer’ associated with a radar sensor and then use the radar measurements to update the kinematic state property buffer of a vehicle being tracked.

One of the main advantages of separating data and computation is decoupling of independent data processing. One can for example use different cores for parallel updating of independent properties associated with the same object. Besides this, the different aspects of a single object are handled by independent pieces of code instead of being part of the same class, e.g., advanced behavior prediction and shape estimation can easily be separated into different components even if they both relate to vehicles.

#### 4.2.4. Interface and Output

Different applications require different subsets of world model data. For a lane-keeping assist application the positions of the lines of the ego-lane are important, whereas adaptive cruise control requires kinematic information from the preceding vehicle. Adapters subscribe to specific property buffers, extract the relevant information and convert it to the format required by the application.

In addition, feature extractors can be associated with adapters that configure the feature extraction algorithm’s parameters, e.g., by setting a region-of-interest or internal parameters, such as thresholds, leading to an improved performance.

## 5. Implementation

The proposed architecture is implemented in C++, and templates and inheritance enforce the constraints that are present throughout the architecture. Open source software is adopted in different parts of the code. ROS [68] is mainly used for visualization, data recording, drivers and compilation. OpenCV [69] is used for image processing and calibration of cameras, whereas the Point Cloud Library (PCL) [70] is used for representing and manipulating LiDAR data. The majority of estimation algorithms is built on top of the Bayesian Filtering Library (BFL) [71] that offers a framework for implementing Bayesian filters and the required system and measurement models.

Different components (modules) are implemented as plugins that are configured using yaml files. The interconnection of different plugins into one multisensor data fusion framework can be performed using the same yaml file. Listing 1 shows a simple example of a YAML file that loads two plugins, each with a different name and associated with a library. Each plugin is associated with a list of optional parameters. The first plugin defines the vehicle model. It defined relative positions of the sensors on an entity with unique identifier prius that is provided as the input argument together with the path to the vehicle model. The second plugin facilitates integrating laser scanner data into the world model. It checks for new data on a ROS topic and adds available measurements to the laserscan_measurement property of the entity with identifier prius. It is good to keep in mind that all applications that will be presented in Section 6 use the same framework. Besides application-specific algorithms, the configuration file is the only thing that has to be modified.

Listing 1 **Example of a simple yaml configuration file.**

plugins:
  # Load the model the car
  - name: load_model 
   lib: libed_load_model_plugin . so
   parameters:
    model_paths:
     - path: /path/to/model
    model_names:
     - type: prius
      id:  prius
  # Laserscan integration
  - name: laserscan_ros 
   lib: libed_ros_laserscan_plugin . so
   parameters:
     topic: /prius/io/lidar/scan
     entity_id: prius
     property: laserscan_measurements

**Figure 5:**
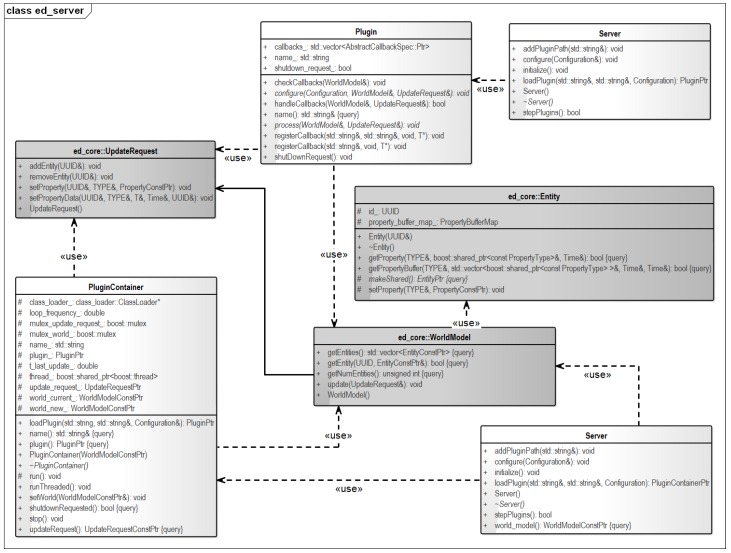
UML diagram of the world model server.

It is not feasible to give all details of all software packages; therefore, this section only explains the core components in more detail. Figure 5 shows a simplified UML diagram of one of the main components in the software architecture. The world model object (roughly in the center) contains the entities that together form the world model. Each entity (shown above the world model block) has a unique identifier and a set of associated property buffers, as explained in Section 4. Manipulation of world model data is performed by plugins (on top) that are triggered using a callback mechanism. Plugins take world model data as an input and compute an update request (left). The update request contains the proposed changes, e.g., newly-added objects or sensor data, updated state estimates, and is processed by the world model object. It can involve an arbitrary number of entities and property buffers. Notice that apart from plugins, there are plugin containers (lower left). The containers take care of the administration, whereas the plugins manipulate the world model. Finally, there is the world model server (upper/lower right) that parses the yaml file and loads and configures the plugins.

**Figure 6:**
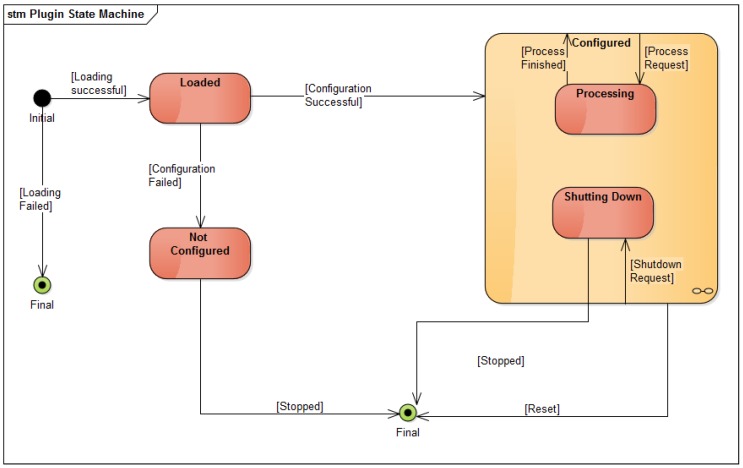
UML diagram showing the state machine underlying each world model plugins.

In order to give more insight into the working of plugins, Figure 6 shows the state machine associated with plugins. First, plugins are loaded and configured by the server. In case the plugin is launched successfully, the callback mechanism triggers a function that takes world model data and processes these data. The result is fed back to the world model. The conceptual difference between the input, interface and estimation modules in Figure 4 is less visible at the implementation level, since each one of these modules is a plugin that interacts with the world model.

In case one vehicle runs multiple applications, one may instantiate one instance of the proposed software architecture for all applications together. The biggest drawback is the single point of failure: a failure in the world model may affect all applications. It on the other hand allows for interactions between the different entities and sharing world model data and computations across applications.

In the presence of contradicting requirements on, e.g., the interfaces, data types, latency or platform that run the algorithms, one may as well launch multiple differently-configured instances of the same software and as a result construct fully-independent world models. Unique identifiers are only guaranteed to be unique within each world model, and similar or identical computations might be repeated in different instances. On the other hand, the conflicting requirements can be dealt with, and the world model no longer is a single point of failure.

Adopting hybrid solutions, where some applications share a world model and other applications have independent world models, is straightforward and does not require any changes in the methodology or software architecture. This will be the most realistic setting in case the number of applications increases and safety-critical applications are involved. Regardless of the configuration, each world model can perform the estimation of independent properties and sensor data processing distributed and in parallel if preferred. This speeds up computation and is advantageous from a fail-safe perspective. Instances of the software have been run on desktop computers, laptops and embedded platforms. Although it is not explicitly mentioned, existing solutions [33,50] allow for running different instances of the same code. However, these solutions lack flexibility in terms of changing interfaces or settings.

## 6. Applications and Results

This section has two goals. First of all, it shows how to work with the proposed methodology. Secondly, it illustrates how the methodology concretizes the accompanying software architecture. This is illustrated by applying the methodology and the software architecture to three example applications that are considered realistic for the domain of automated driving. The examples are diverse in terms of objects involved, sensors, required interfaces and algorithms. The only effort required when switching applications is (i) reconfiguration of the software architecture explained in Section 4 and Section 5 and (ii) the implementation of application-specific algorithms and models (if any). None of the existing solutions found in the literature would be able to serve this diverse set of applications without fundamentally changing the design of the software from one application to the other.

1. Example 1 demonstrates low-level sensor fusion of data from multiple radar sensors using nonlinear models. The combination of sensors enables estimating quantities that none of the sensors could have estimated individually.
2. In Example 2, different sensor modalities with different interfaces are combined. Information from one sensor is used for configuring another sensor’s feature extraction algorithm.
3. The traditional approach as shown in Figure 1.

Notice that we will be short on technical details since the focus is on demonstrating the flexibility and reusability of the proposed methodology and software architecture in different applications rather than the technical aspects and performance of individual algorithms.

### 6.1. Example 1: Vehicle Tracking and Shape Estimation

Application-dependent output selection: The goal for the first example is tracking the position and velocity of vehicles on a highway and simultaneously estimating their length. Typically, such information is useful in platooning applications [2], including automated lane changes and splitting and merging platoons. So far, the application does not exploit probabilistic information.

**Figure 7:**
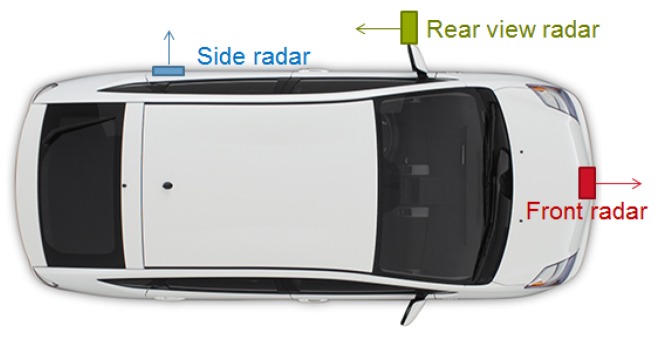
Three radars used for simultaneous vehicle tracking and shape estimation.

Sensors: Radars were used mainly because of their robustness to environmental conditions and the availability of commercial automotive-grade radars. The locations of the sensors on the vehicle had to be feasible for passenger cars, as well as trucks. A three-radar configuration was adopted as shown in Figure 7: one radar on the front bumper of the host vehicle, one radar on a mirror (looking backward) and one radar on the rear left door (looking sidewards). The radar on the side is only able to detect the presence or absence of objects and is typically used for automatic door control. Notice that, as usually is the case, many application-specific constraints play a role in the sensor selection and, therefore, constrain the space of world modeling solutions.

For this application, the world model will contain three radar sensor entities, an arbitrary number of entities representing the other road users and the host vehicle entity. The property buffers associated with these entities include radar detection buffers, estimated vehicle state buffers and the relative positions of the sensors. The number of consecutive measurements and time without state update are used for object existence models.

**Figure 8:**
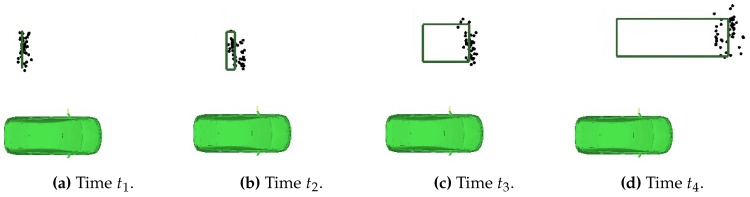
As a vehicle (possible positions in black) overtakes the host (in green), its length is recursively being refined (rectangle), here $t_{1}<t_{2}<t_{3}<t_{4}$.

Figure 8 shows how the combination of an estimated position and velocity enabled by the caravan mirror radar and the presence/absence detection of the side radar facilitates estimating the length of overtaking vehicles: the front bumper position is being propagated while the length is updated as long as the overtaking vehicle is detected by the side radar. In order to allow for this geometry estimation, a feature extraction module is added. This module takes as input a predicted position of the vehicle’s center and calculates the minimum length of the vehicle in case the side radar detects the presence of this vehicle. More details about this application and technical details can be found in [32]. None of the architectures in Section 2 support using this sensor set due to interface requirements, and therefore, it is not possible to reproduce these results without changing the architecture of these works.

State: The state contains all of the desired outputs (vehicle position, velocity and length) and is represented by a Monte Carlo probability density function.

Adapter: The adapter takes the expected value of the probability distributions representing the vehicle states. No probabilistic information is accepted by the application.

Data association: For the side radar, measurements are associated with any object near the sensor. The caravan mirror and front bumper radar measurements are associated with state estimators by solving the bipartite graph version of the Kuhn–Munkres Hungarian method [72].

Bayesian filtering: The radars are configured such that they deliver low-level data. As a result, the fusion can either be performed by a single Bayesian estimator using the low-level data or at a higher level in case each radar will be associated with its own estimator. In the latter case, an additional fusion step that combines the outputs of all single sensor estimators is required.

For this application, the first of the options is preferred, since otherwise it is not possible to perform the estimation of the state. In other words, performing low-level fusion enables estimating quantities that could not have been estimated by any of the sensors individually.

The motion model used for tracking vehicles assumes a constant velocity. To compensate for possible accelerations, the system model noise is related to expected acceleration and decelerations, as explained in [65]. Objects are assumed to not change shape.

The estimated vehicle length is used to map the position of the vehicle center to the position of the front or rear bumper as measured by the backward- and forward-looking radars. The linear measurement models are associated with Gaussian and zero-mean measurement noise. The side radar requires a more advanced measurement model mapping a predicted vehicle position and size to a Boolean measurement. This measurement model furthermore incorporates a spatial constraint (an overtaking vehicle cannot overlap with the host vehicle). The measurement model for the vehicle length estimation is linear and associated with zero-mean Gaussian measurement noise.

**Figure 9:**
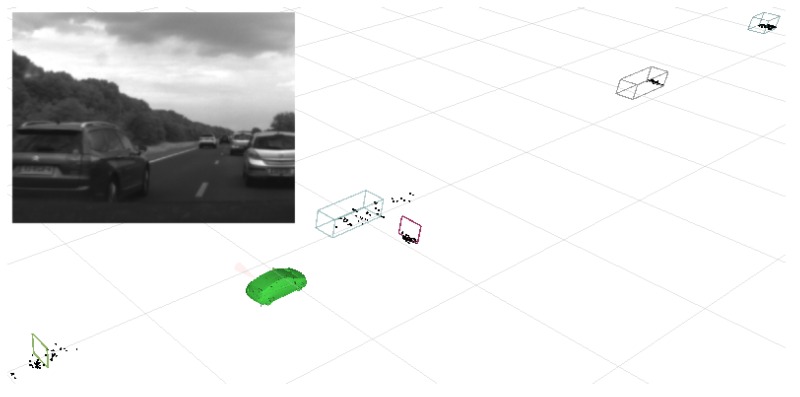
World model impression. Vehicles that have overtaken the host are associated with both kinematic and geometric information, whereas other vehicles have an unknown geometry.

The nonlinear side radar measurement model enables the length estimate, but complicates using Kalman filters and Gaussian probability distributions as the interface. The main reason for selecting a particle filter for state estimation is its ability to facilitate such advanced measurement models. Marginalizing out the geometry enables solving the geometry estimation analytically and thereby reduces the size of the state space from which particles have to be sampled. This is why a Rao-Blackwellized particle filter was selected. More technical details can be found in [32]. The resulting world model while driving on a highway is shown in Figure 9.

### 6.2. Example 2: Line Detection

The second example uses a different sensor set, estimates different quantities and demonstrates the reconfigurability of the software architecture.

Application dependent output selection: The proposed output for this example is the shape of the lines on the road, represented by second order polynomials. The envisioned application is lane keeping on highways.

Sensors: One forward looking camera is used for detecting the lines. Besides these 360 degrees, LiDAR data are available from a series of 2D laser scanners.

**Figure 10:**
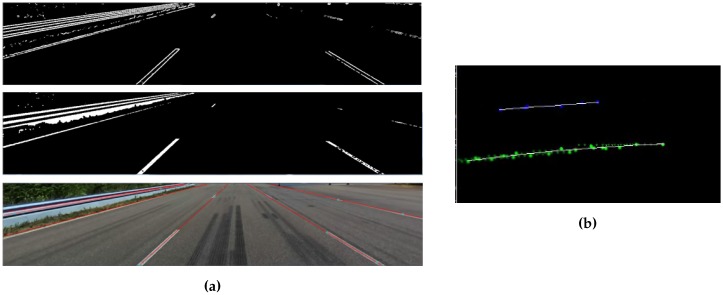
Camera processing steps. (**a**) Feature extraction, first adaptive thresholding delivers edges (above); then using the Hough transform, lines are fitted (middle). The result is shown in the lowest figure. (**b**) Second order polynomials (in white) are fitted through all line points (blue left lane marking, green: right lane marking) detected in the last 20 m.

Feature extraction is added for the camera images since the raw camera images are too complex to feed to our estimation algorithms directly. First, adaptive thresholding aims at finding edges that might represent lines, then a Hough transform fits straight lines. These steps are shown in Figure 10a. In order to get a second order polynomial from a collection of straight lines, all lines in the last 20 m are combined in a top view image, and second order polynomials are fit (see Figure 10b). No tracking is needed, since this line aggregation is a form of temporal filtering. This approach typically delivers the smooth results that are desired for the application. In case more advanced feature extraction is preferred, e.g., deep learning [63], such algorithms can be used to replace the proposed line detection algorithm without redesigning the software.

To lower the number of false or inaccurate line detections, the line detection algorithm will use a region of interest based on the road edges detected by the laser scan data. Only lines compatible with the road geometry are considered valid.

State: Lines will be represented by second order polynomials; hence, three coefficients will be estimated for each of the lines detected: the lane’s curvature, heading and offset. All information should be represented with respect to the host vehicle. Lines can be associated with a confidence based on the quality of the fitted polynomial.

Besides lines, the road edges will be estimated from the laser scan data. For this purpose, first, a map is constructed, and then, the edges will be extracted. Apart from the lines, the world therefore contains a map that is processed independent of the lines.

Data association: For this application, the line aggregation modules needs to associate lines with previously-observed lines. This association is performed using a basic distance-based threshold.

Adapter: The adapter extracts the lines from the world model and provides them to the application.

Bayesian filtering: As indicated before, the lines are not tracked by a Bayesian filter. The map however does require Bayesian estimation. The prediction step compensates for the motion of the vehicle; the update step allows for adding new measurement data.

**Figure 11:**
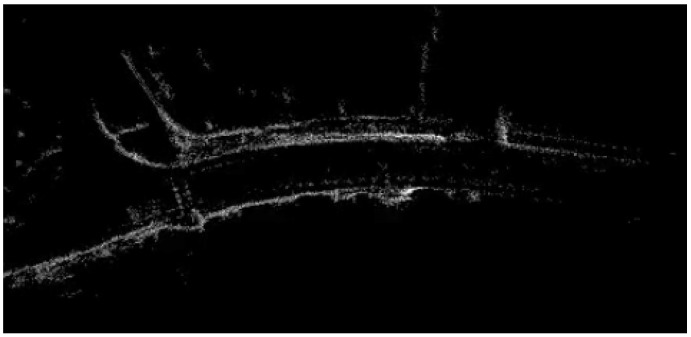
Map used for road edge detection.

In the prediction step, the car’s odometry data are incorporated to compensate for the motion of the vehicle. Integrating odometry information leads to drift, e.g., due to wheel slip, and therefore, leads to blurry road edges in the map. By adopting a Rao-Blackwellized particle filter [21] that simultaneously estimates both the map and the host vehicle’s position within this map, the effect of drift is reduced, and sharper road edges are obtained. Then, the measurement simply adds new points to the map. Figure 11 shows an example map that is obtained this way.

To keep the map compact, a sliding window is used to remove faraway parts of the map, and the point cloud map is downsampled after every update.

Compared to the estimation in Example 1, only the measurement model and the state representation of the Rao-Blackwellized particle filter have changed, and the image processing was added. Due to the flexibility of the software architecture, the vast majority of estimation code could be reused. The code related to configuration of the world model, loading models, adding sensor data and inspecting the performance was kept the same.

### 6.3. Example 3: Bicycle Tracking

In the last example, the software architecture is used to implement the traditional pipeline as shown in Figure 1. Furthermore, it deals with a different type of objects and is the only one of the examples that could have been implemented in case any of the multisensor data fusion architectures explained in Section 2 would have been adopted.

Application-dependent output selection: In the last example, bicycles are tracked.

**Figure 12:**
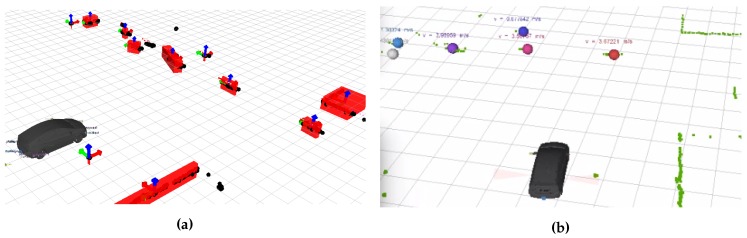
Bicycle tracking using the proposed software architecture and configured according to the traditional world modeling pipeline. (**a**) Features (in red) extracted from the LiDAR data (in black) before removing bounding boxes that are not compatible with typical bicycle dimension; (**b**) the host vehicle (in black), the LiDAR data (in green) and the tracked bicycles (colored spheres).

Sensors: Detections are performed using 360${}^{\circ }$ 2D LiDAR data. These data are clustered, and bounding box features are extracted; see Figure 12a. Only bounding boxes that are compatible with typical bicycle dimensions are kept. Adding radars that are present on the vehicle and delivering object track level data are straightforward, but are left for future work.

State: The state is represented by a Gaussian distribution and contains the bicycle’s 2D position and velocity.

Adapter: The adapter selects the bicycles from the world model and takes the expected value of the Gaussian distributions.

Data association: Data association is performed using the algorithm explained in Section 6.1.

Bayesian filtering: An extended Kalman filter is used for tracking due to the nonlinear measurement model that compensates for ego motions of the host vehicle. The linear measurement model is associated with Gaussian noise for position measurements. The velocity is not measured. The resulting output is shown in Figure 12b.

Adding radars as suggested in Step 2 of this example can be done by adding any track-to-track fusion plugin that enables processing measurement with unknown correlations in a fixed network topology.

## 7. Conclusions and Recommendations

A one-size-fits-all multisensor data fusion approach is unrealistic due to the large diversity in applications, vehicles, requirements and sensors. Ignoring this diversity and adopting a single world modeling algorithm at best requires redesigning the algorithm on a regular basis. However, it may as well disable deploying applications due to contradicting requirements on, e.g., the estimation accuracy, smoothness or latency associated with the one-size-fits-all world model.

As an alternative, this work has introduced both a multisensor data fusion methodology and a flexible software architecture for automated driving applications. The combination is optimized towards reconfigurability and minimizes the required effort in case sensors, requirements or applications change. The methodology suggests families of algorithms and identifies constraints between various design decisions, thereby speeding up the process of algorithm selection. This result should lead to more effective world modeling in the vibrant and dynamic field of automated driving applications.

The proposed methodology and software architecture are applied to three different real-world examples. These examples demonstrate the work flow associated with the methodology and the reconfigurability of the proposed software design. None of the related works allows for the diversity in applications, and this work therefore is seen as a first step towards a more effective way of sensor data processing and fusion for automated driving. Vehicle position and velocity tracking and simultaneous geometry estimation on highways (radars), road marking detection (LiDAR, camera) and position and velocity tracking of bicycles in an urban environment (LiDAR) could be realized by reconfiguring the multisensor data fusion framework and implementing a minimal set of sensor and application-specific algorithms.

In the near future, we will continue to apply the results in this work to a diverse set of applications and on different vehicle types.
